# The challenges associated with the prevention of smuggling and counterfeiting health goods in Iran

**DOI:** 10.1186/s12889-024-18637-0

**Published:** 2024-06-11

**Authors:** Farzaneh Mahmoudi Meymand, Amirhossein Takian, Ebrahim Jaafaripooyan

**Affiliations:** 1 Department of Health Management, Policy, and Economics, School of Public Health, Tehran University of Medical Sciences, Tehran, Iran; 2 Department of Global Health & Public Policy, School of Public Health, Tehran University of Medical Sciences, Tehran, Iran; 3 Health Equity Research Center (HERC), Tehran University of Medical Sciences, Tehran, Iran; 4 Department of Health Management, Policy, and Economics, School of Public Health, Tehran University of Medical Sciences, Tehran, Iran

**Keywords:** Policy analysis, Prevention, Medicines, Counterfeit, Pharmaceutical products, Smuggling, Iran

## Abstract

**Background:**

Smuggling health goods given the importance and critical nature of health services should be undeniably addressed and controlled by all countries. This issue is especially more widespread in developing countries with more damaging consequences. This paper therefore aims to identify and analyze the challenges of preventing smuggling of health goods in Iran.

**Method:**

Within this qualitative study, we conducted face-to-face, semi-structured interviews with 30 purposefully recruited key informants and stakeholders in the detection, prevention, and combating of health goods smuggling. Each interview was analyzed thematically, using an inductive approach to generate codes, then categorized and presented in the form of main themes and sub-themes. Maxqda 11 assisted in coding, analysis, and data management.

**Results:**

Three main themes emerged representing the challenges of prevention of smuggling in Iran in the areas of anti-smuggling policy development, including categories of inefficient policy and plan, and failure to reach agenda; policy implementation; categorized into actors, resources and instruments, and implementation guarantee; and finally monitoring and evaluation; including, procedures and practices, and the role of surveyors.

**Conclusion:**

Prevention of smuggling health goods proves to be a highly complex, challenging, and multi-faceted practice. Therefore, strengthening policy-making, regulatory frameworks, and facilitation functions about smuggling, counterfeiting, and corruption should be promoted in parallel.

**Supplementary Information:**

The online version contains supplementary material available at 10.1186/s12889-024-18637-0.

## Background

 The pharmaceutical market is considered potentially as the third largest sector of the international black economy, after the firearms and drug markets. The World Health Organization (WHO) experts estimate the total value of the global illegal pharmaceutical market up to 30 billion dollars [1]. Given their global spread, the smuggling and counterfeiting of pharmaceutical goods are now seen as an international (transnational) phenomenon. Smuggling is the process of moving goods or products illegally into or outside a country in response to an unmet need. This may occur to compensate for a deficiency or in an effort to maximize specific profit [1, 2]. It has allegedly widespread damaging effects in terms of economic, social, cultural, political, and security aspects, and in a way, may give the way to more corruption. As to health sector, it could be a serious threat to the lives of consumers, due both to the supply of fake and counterfeit medications and the inappropriate storage and distribution conditions [3].

Over the past decade, an increasing number of countries have reported counterfeit medicines (falsely labeled), while the types and amounts of counterfeit medicines being distributed have significantly increased [4]. The case is more serious in low and middle-income countries (LMICs), where the cost of legal medicines might be unaffordable for some people and legal controls are often poor [5]. WHO has been warning about the impact of counterfeiting and counterfeit medicines on consumer trust in healthcare systems, health professionals, supply chains, and genuine suppliers of medicines and medical equipment. It has further reported that counterfeit medicines potentially account for over 50% of the global medicine market and that a significant portion of these fake products are discovered in the LMICs [6]. Accordingly, In 2012, WHO launched its Global Surveillance and Monitoring System (GSMS) for substandard and falsified medicines aiming to improve the quality of reporting, data usage to inform the market, and building the regulatory capacity [7].

Whilst around one-third of the world’s population lacks timely access to high-quality medicines, the estimates suggest that at least 10% of medicines in the LMICs are substandard or counterfeit, costing approximately US$31 billion annually [8]. The COVID-19 pandemic has also given a rise into the distribution of counterfeit products, especially in the LMICs [9]. Some reports estimate that 80% of the medicines seized by the customs of Middle Eastern and East Asian countries are counterfeit. It is estimated that between 1% and 70% of the medicines in different countries might be counterfeit [10], such that the statistics are 1% in high-income countries and from 10 to 30% in the LMICs [11, 12]. It is increasing in other countries in the same proportion.

The phenomenon of medicine smuggling in Iran has been happening in two ways: import and export, especially to Iraq and Afghanistan [12]. Iran is apparently a country of incentives and opportunities for smuggling and buying contraband. Various economic, cultural and social, political, situational and legal factors in Iran have somehow created challenges and limitations for confronting and preventing smuggling [13]. Some statistics report the volume of counterfeit medicines over 1% and cosmetics up to 50% in Iran, which was 10–15% before the implementation of the medicine authenticity system [12]. The share of contraband health goods in Iranian market is estimated up to 90% for some items, while less than 10% of these goods are only discovered [12]. Stimulants, abortion pills, sexual stimulants, narcotic drugs, chemotherapy drugs, body beauty drugs, medicines for special patients, energizing drugs, psychotropic medicines, and medicines with restricted import are usually more important to smugglers in Iran [2], whose distribution takes place normally both inside and outside of official networks. Moreover, approximately 80–85% of smuggled medicines are counterfeit. They are imported mainly from Dubai, Turkey, and Pakistan [12]. Import of low-quality, non-genuine, and smuggled medical equipment to medical institutions has the greatest effect on increasing the probability of injuries [14].

Although it is hard to determine the main cause of buying and selling illegal medicines and smuggling, the decisions and actions of the government could be crucial [15]. Human resources, equipment, and laws and regulations were found the most influential factors in preventing smuggling in Iran [16]. According to the law passed by the Iranian parliament in 1955 and its subsequent amendments, the import, export, production, distribution and supply of all pharmaceutical and biological products require a license from the Ministry of Health (*MoH*). In this law, there was no explicit reference to the crime of smuggling medicines except for under the Article 27 of goods and currency smuggling law, approved and enacted in 2013. Considering medicines as goods, this law introduced their smuggling as a general economic crime without specifying explicitly that trafficking counterfeit medicines should be criminalized independently [12]. National regulatory authorities are expected to guarantee access to medicines and combat falsified medical products. Despite the recent advancement, the regulatory capacity in the LMICs, including Iran, is still insufficient for the implementation of value-added monitoring practices and the utilization of evidence-based data to support monitoring actions [8].

Given the critical features of health sector such mainly as life-related outcomes, information asymmetry and externality, a broad range of risks and the volume and variety of vulnerable people could be exposed to the entry and supply of counterfeit medicines into the country or their export to other countries [15, 17]. Therefore, attention to the importance of preventing the smuggling health goods could be highly prominent requiring, especially, support measures in the policy-making process and workable political action. A correct understanding of this issue makes it possible to mitigate its undesirable and inappropriate public health consequences. Present study thus aims to identify the challenges of preventing the smuggling and counterfeiting health goods in Iran.

## Methods

### Study design and sampling

For this exploratory qualitative study the researchers recruited 30 key informants and stakeholders in such areas as detection, prevention, and combating health goods smuggling using purposive and snowball sampling considering maximum diversity (Table 1). We used a generic interview guide, following the pilot-test and modification, for face-to-face, semi-structured interviews (Appendix 1). The interviews were conducted from May to January 2022 and lasted about 80 min on average. During this period, the fear of contracting COVID-19 had decreased in comparison to the previous year, but despite this, preventive behaviors and considerations were observed in face-to-face meetings and participants were free to choose their mode of participation. A file containing the objectives of the study, ethical considerations, a profile of the researchers, and their contact information was sent to the participants with the assurance of confidentiality and anonymity. All interviews were conducted and analyzed in Persian; 14 interviews were digitally recorded and transcribed verbatim. 16 interviewees did not allow recording, for which we took notes. Data collection continued until we reached saturation, until no new data emerged.

#### Participant characteristics

A total of 30 people, including 28 men and 2 women, were interviewed (Table 1).

**Table 1:** The characteristics of interviewees

characteristics		N (%)	Workplace	No
Gender	Men	28(93.3)	Central Headquarters to Combat the Smuggling of Goods and Currency Government Punishment Organization Customs Ministry of Industry, Mining and Trade Economic Security Police University of Medical Sciences Organization for Collection and Sale of State-owned Properties Broadcasting Organization The Economic Commission of the Islamic Parliament Ministry of Health researcher in the field of law and criminology Pharmacist pharmaceutical syndicate Insurance Organization economic activists in the medicine industry	9 2 1 1 2 4 1 1 2 2 1 1 1 1 1
	Women	2(6.7)		
Age	30–35	2(6.67)		
	35–50	16(53.33)		
	50≤	12(40)		
Educational background	Master’s degree	4(13.33)		
	MD &Ph.D. degree	26(86.67)		
Years of experience	5–10	2(6.67)		
	10–15	17(56.67)		
	15≤	11(36.67)		
Participants’ background	A mixture of policymakers, health system managers, Faculty members, Lawyer, specialists, Executive consultants, No organizational affiliation			

### Data management and analysis

Thematic analysis was used to guide the categorization and concept generation from the data through the researchers’ careful examination and constant comparison [18]facilitated by Maxqda11. While the data collection, the coding was ongoing through disassembling and reassembling the data. After coding and a deep reflection on their content, the categories were merged and classified under key themes (Table 2).

### Rigor and trustworthiness

The trustworthiness was ensured based on four criteria proposed by Lincolon and Guba, including credibility, confirmability, dependability, and transferability [19]. Researchers’ long-term engagement with data collection (11 months) and their continuous investigation and review of codes, categories, and themes were as such conducted. The initial codes were returned to 12 interviewees and checked with two experts in line with respondent validation and peer examination. Finally, maximum diversity was ensured in the selection of participants from different organizations. This process ended with all authors agreeing on the final classification after discussion of any discrepancies.

As to the reflexivity; first, the research team, given their background in health policy and research, designed, pilot tested and revised the interview guide before the actual interviews were conducted. Then, during data collection, the interviewer avoided asking leading questions and directing the participants’ answers in order to minimize the potential bias, and presented the general impression of what they said, subsequently to the interviewees. There were no conflict of interest between the interviewees and the researchers. Ultimately, before reporting, the findings were checked by peers to ensure that they accurately reflect the views and experiences of the participants, not the researchers.

## Results

Several challenges were identified for preventing the smuggling and counterfeiting health goods in Iran, classified into three main themes and seven categories including mainly; formulation /development, implementation and monitoring and evaluation of prevention policies (Table 2).

**Table 2:** Challenges towards prevention of smuggling and counterfeiting health goods

Theme	Category	Codes
**Formulation/ development of anti-smuggling policies**	**Inefficient Policy and Plan**	• Ineffective/inefficient prohibition and restriction policies on health goods imports • Confusion and ambiguity and contradictions in the related laws and regulations • Parallelism and friction in policy making and planning • The weak role of the private sector and NGOs in policymaking
	**Failure to reach agenda**	• Low political priority for anti-trafficking programs • Lack of policies for newly established pharmaceutical companies • Poor support for development policies of commercial diplomacy • Failure to develop and implement anti-monopoly laws • Low access to appropriate educational content
**Implementation**	**Actors**	• Low power of law-enforcing bodies • Failure to select and appoint capable and committed executives • Organizational and personal conflict of interests • Lack of trust between government and business actors, planners, and executives • Consumer low confidence in the quality of domestic products
	**Resources and instruments**	• Inefficient motivational system • Multiplicity/fragmentation in decision-making and executive bodies • The complexity of the supply chain of health goods • Poor supply chain performance of health goods • Lengthy, complicated licensure issuing process
	**Policy Implementation Guarantee**	• Non-alignment of upstream plans and documents with the operations • Threats by the supporting measures of neighboring countries • The effect of sanctions • Variations in the managers and authorities • Golden signatures • Lack of economic stability
**Monitoring and Evaluation**	**Procedures and practices**	• Lack of a comprehensive information and statistical system • Lack of information transparency • Lack of systematic attitude in the monitoring process • Complexity of monitoring and evaluation • Lack of equipment for advanced electronic monitoring • Difficulty in monitoring advertisement and promotion of products • Ignoring the regulatory role of other responsible institutions
	**Surveyors**	• Failure to select and appoint capable and committed checkers • Insufficient/ workable incentives

## Formulation /development

For a variety of reasons, our laws are not deterrents and the direction of the established policy is not seemingly effective, according to most of the managers interviewed in this study. Several challenges indicating the lack of formulation and development were revealed under two main areas of Inefficient Policy and Plan, and Failure to Reach Agenda.

### Inefficient policy and plan

#### Ineffective/inefficient of prohibition and restriction policies on health goods imports

The adoption of a policy of banning and restricting imports is one of the roots of smuggling in the country: “*We have three groups of goods; The first are banded goods, which have no legal market in Iran because there is a market for them; they are always smuggled, such as abortion medicines and… prohibited goods in the economic sense are the second category. For reasons such as supporting domestic production, a lack of foreign currency, and embargoes, we have banned their imports. If there is a market for the product, banning is pointless. Goods that are not necessarily banded but have high import tariffs fall into the third group. For example, we have always seen this in the case of food supplements, some medical consumables, and some laboratory consumables such as kits” (*p1, 2).

The preparation of programs is more for the satisfaction of the authorities and the aspect of efficiency is less taken into account. One participant said: “*Some existing policies needn’t apply. For example, this directive is 90% additional, and this creates implementation and exchange costs. Policies should be skill-based too” (*p14). Another interviewee said: “*The policy formulated by the Ministry of Health is a sledgehammer, and it is questionable whether these policies will lead to results!!*” (p7).*”*

#### Confusion and ambiguity and contradictions in the related laws and regulations

Confusion and ambiguity in laws and regulations can provide the context for irrelevant interference, uncertain decision, and the possibility of abuse: “*The content of the laws is problematic; in some of them, the possibility of interference is high, i.e., the policy was initially well-intentioned in its formulation, but in its implementation it has been acted upon differently. Sometimes* writing vaguely *provides a context to cover many issues”* (p4).

One interviewee complained about the policy of granting many exemptions and saw the dual treatment of trafficking as resulting from the contradiction in the current laws. He said: *“Kolbars and sailors have a quota to bring in smuggled goods, and this is legal. Passengers going to Turkey use this platform as an opportunity to enter the country with contraband”* (p13).

#### Parallelism and friction in policy making and planning

We have and should have interdepartmental cooperation; it is reflected in this Electronic Packing List (EPL) system, which has approximately 25 member organizations in it, and it is necessary that this cooperation and interaction goes beyond that. *“An integrated system should be designed, and the information should be entered in one place in an integrated manner, so that there is no duplication”* (p2).

#### The weak role of the private sector and NGOs in Policymaking

Interviewees acknowledged the importance of the role and position of non-state elements in policy-making: “*If the private sector and associations and institutions were given more opportunities to participate in policymaking, the situation would be better, but they’re practically ignored”* (p7, p10). *Another said: “If the government wants to move forward and be successful, it should serve the private sector because it drives the cycle of the economy, or at least part of it”* (p2).

### Failure to reach agenda

#### Low political priority for anti-trafficking programs

Examples of the low political priority given to preventing smuggling by politicians and government managers include the failure to appoint a special presidential representative to monitor smuggling, how the relevant budget and credits are spent, and resistance to the introduction of preventive legislation: “*The chief of staff is not a special representative whose signature is the president’s signature*. *The priorities of the fight against smuggling are clear, and they get a plan, a line, and a budget around it, but because they do not believe in this plan, they do not implement it” (p3).* Another participant pointed out, “*How many times was this law (an anti-monopoly law to prevent smuggling) proposed to parliament, but parliament did not even consider it, and the first agenda was blocked?*” (p4).

#### Lack of policies for newly established pharmaceutical companies

Government support for start-up pharmaceutical companies was seen as crucial. As one participant comments: “*Some countries, to support the domestic producer, do not include the general control price in the total price of the drug produced by the* start-up *company for the first year… even in the case of recombinant medicines… This is to prevent monopoly; companies should be encouraged to produce new drugs, and research and development (R&D) should be strengthened, but our governments do not provide constructive support to* start-up *drug companies”* (p7).

#### Poor support for development policies of commercial diplomacy

The development of international trade through support for the acquisition of brand representation for commercial companies and the need for attention to and compliance with modern trade laws in the field of commercial diplomacy is important. According to one of those interviewed: “We’ve got *the wrong policy because we does not have an official representative of some specific brands in the country. if there is a demand for that brand in society, the buyer more or less turns to it, and if he can’t get officially, he turns to smuggling to get it… and we see that in informal distribution networks”* (p8). According to another, “*Unfortunately, our market is governed by custom, not modern business rules”* (p9).

#### Failure to develop and implement anti-monopoly laws

The importance of drafting and implementing an antimonopoly law in Iran to prevent the formation of monopolies in the healthcare market and to ensure a competitive market was raised. *“We have no anti-monopoly or antitrust law in Iran. The anti-monopoly law says that you do not have the right to own more than 50% of the market… to prevent monopolies from forming and to maintain economic competition, there must be others”* (p18).

#### Low access to appropriate educational content

Sometimes the production of educational content and advertisements leads to the creation of needs in the audience and is not in line with the country’s policy in the field of dealing with consumerism, and sometimes the production of content in the field of identifying and disadvantages of smuggled goods is weak and ineffective. One interviewee commented: “*We need to prevent the promotion of consumerism in society, and this issue has led to limitations in advertising, awareness raising, and producing educational content for some products, especially cosmetics; conversely, some interested organizations fail to produce and publish educational content”* (p29).

## Implementation

Managers and policymakers need to have a good understanding of the environment, structure, behavior of actors, and processes to align their programs, policies, and services to the needs and expectations of society. The main examples in this section are grouped under three themes: Actors, Resources and instruments, and Policy Implementation Guarantee.

### Actors

#### Low power of law-enforcing bodies

Politicians are unaware of the importance and role of law enforcement in decision-making and policy-making positions. An interviewee raised: *“The organization called “*CHCSGC*” with several ministers does not have and cannot have the necessary efficiency, because it does not have the executive power and* authority to give orders to the Ministers*”* (p16).

#### Failure to select and appoint capable and committed executives

Some respondents emphasized the policy of appointing those with executive responsibility: *“It’s very good that the policies are reviewed, but let’s also establish a policy for the person who is going to the executive. He is the one who performs the implementation of the policy, the writing of the instructions, or the evaluation. Do not leave them alone and deal only with this paper text of the policy. Even if the policy is written correctly but the executor is not a righteous person, he can interpret it, and give justifications while doing wrong” (p4).* Another added: *“The software and the hardware systems are important, but the* skills and knowledge of *person who is sitting behind the network is important as well” (p12).*

#### Organizational and personal conflicts of interests

Sometimes, natural or legal persons suffer from the problem of conflict of interest because of their position in the government (personal conflict of interest), and sometimes the institutional structures of the government are designed in such a way that any person in that position is exposed to conflict (organizational conflicts of interest). One of the interviewees stated: *“The FDA is responsible for pricing, supporting consumer purchasing power, protecting insurance company interests, and trying to keep prices low while ignoring pricing requirements. These multiple roles have led to the FDA not being able to act successfully in terms of organizing demand, making effective policy decisions, and monitoring the production of medicines”* (p23). One interviewee explained the existence of the conflict of interest in the government policy cycle: “*The conflict of interest can show up in the agenda-setting stage and not allow politics to get on the agenda at all; now, if it gets past that stage, in the formation stage, it becomes mischief, and usually the people involved formulate policies in such a vague and two-sided way that later, in the executive bodies, it is up to them to decide how to implement them… Finally, there can be conflicts of interest at all these stages“(p24).*

#### Lack of trust between government and business actors, planners, and executives

The role and importance of stakeholders as individuals, groups, and organizations with interests and potential to influence an organization’s action, goals, or policy direction is clear. One of the interviewees stated: *“Because of their mental background and lack of trust, the view of commercial activists in distribution networks is different from any policy adopted by the government and parliament. It is for this reason that they do not apply a guidelines” (*p26). Someone else added to it: “There *is a mutual lack of trust between planners and managers in the country”* (p15).

#### Consumer low confidence in the quality of domestic products

The lack of confidence in the quality of the domestic product leads the consumer to buy foreign smuggled goods, and sometimes there is no economic justification to go for domestic production. Person stated: *“People are hesitant about the quality of domestically produced health goods and do not believe in their effectiveness; misinformation and misconceptions can be difficult to eradicate”* (p28).

### Resources and instruments

#### Inefficient motivational system

The interviewees spoke about the motivation system and the need to pay attention to the internal and external motivation of government employees. As one participant put it: “*If you give an employee a dollar as a bonus, extrinsic motivation, and on the other hand work on the employee’s commitments and beliefs, internal motivation, he won’t get the illegal $9, and won’t enter the cycle of rent-seeking and corruption in bringing in and taking out illegal goods” (p17).* Another interviewee stated: *“In our country, the incentive system is not valued; promises are made but not kept; right of discovery has a name, but it is like heaven! (Allusion is to nonpayment or late payment* of royalties)*” (p13).*

#### Multiplicity/fragmentation in decision-making and executive bodies

The multiplicity of decision-making and procedural bodies, the lack of integrated management, parallel work, and friction between institutions were among the most important issues mentioned by the interviewees about this issue. To put political content on the agenda, the problem needs to be solved in the complex structure of administrative circulation. An interviewee stated: “*I see a lot of conflicting orders; there are too many decision inputs. When there are too many executive orders and news announcements, it disrupts the implementation process”* (p28). *“When the bureaucracy is defined, the units are not seen separately, they are seen as integrated, but now it has become an island” (p30).* According to the opinion of some of the interviewees, the main cause of many process and structural problems is the FDA’s accumulation of authority and intrusion into many unrelated work areas. One of those interviewed said: “*The FDA is just a regulatory and rule-making body that is all; we’ve given the FDA too much power” (p9).*

#### The complexity of the supply chain of health goods

Long and complex supply chains make it easier to smuggle and commit fraud. An interviewee said: “*It is important that the process are simple, transparent to the business operators and that the changes are very small when the systems are designed and implemented. Unfortunately, this is not happening”* (p7). Another interviewee added: *“The published reports are not smuggling; they are off-grid supply. It is the sale of hoarded goods” (*p8).

### Poor supply chain performance of health goods

Irregularity in the production and distribution processes and mismanagement of production schedules were among the issues cited by those interviewed. “*In these stories, our investors are more dependent on the wave than on the logic of the long-term. These irregularities encourage and stimulate the occurrence of smuggling, and sometimes it is intentional…” (*p4). According to another, *“the poor performance of the distribution network in emergencies, unplanned and unforeseen deliveries, pushes people to smuggle”* (p27). The weakness and unprofessionalism of the purchasing system in the pharmacy, the lack of use of modern methods in the identification and tracking of smuggled products, and the delay in the establishment of systems were examples of this theme. One interviewee explained: *“We have a tragedy after these systems; we want to mechanize processes that have not yet been corrected… The weakness and unprofessionalism of the system of purchasing medicines in pharmacies is obvious”* (p3).

#### Lengthy, complicated licensure issuing process

The role of trade facilitation in prevention was mentioned by some interviewees. If we are too strict, perhaps the importing company will accept its risk and permit itself to import goods in the form of smuggling. An interviewee stated: *“In Georgia, a licence for a pharmaceutical company is issued within 12 hours, but in Iran we have to spend at least a year for this….The motto of facilitating trade is given, but in practice, it’s not facilitated, and traders are inconvenienced anyway, and it will rather import the goods in the form of smuggling”* (p30).

### Policy implementation guarantee

#### Non-alignment of upstream plans and documents with operations

The gap between strategic levels (leadership) and operational levels (management) should be considered. One of the interviewees stated: *“Sometimes in our plans and policies we look at an isolated area and we want our entire distribution networks to be properly implemented in this isolated area! Seen in this way, our policies and strategies are fundamentally flawed” (*p19). *According to another, “There are institutions that receive government funding, but we see no link between the policy and the announcements”* (p20).

#### Threats by the supporting policies of neighboring countries

Iran’s smuggling has been affected by the supportive policies of neighboring countries, including Iraq, Turkey, and Afghanistan, in attracting domestic and foreign: *“The country of Iraq welcomes manufacturers and companies that import production line equipment and machinery, and the government gives them exemptions and interest-free loans to carry out production in their country; this can affect the attraction of investment in our country. Most of the foreign investors, or even our domestic investors, are willing to cooperate with the neighboring country”* (p5). “*In the case of Afghanistan, because they don’t have domestic production and there is no serious policy to prevent smuggling of Medical Goods (PSHG), we are witnessing reverse smuggling”* (p6).

#### The effect of sanctions

The imposition of sanctions has an impact on the formation of smuggling networks in the form of an “official network”. “With *official network smuggling, the government officially imports, through several companies, some of the goods it needs but cannot import due to sanctions and international laws, including industrial equipment, raw materials, etc.”* (p4).

#### Variations in the managers and authorities

Changes in the power structure, and subsequent changes in senior management within organizations can be facilitators or inhibitors of the implementation of anti-smuggling policies. One interviewee asked: “*How long is the life of my policies? Suppose they issue 200 notices over two years; the life of each of my policies is not enough for the network to understand it; to understand it, someone else is responsible”* (p21). “*Each government considers the program presented by the previous government and even by the one before it, to be the source of a lot of inconsistencies and shortcomings, and does not feel obliged to carry it out” (*p15).

#### Golden signatures

There is a lack of serious support for accountability, implementation, and evaluation of relevant policies because of the existence of some exceptions due to the golden signatures of white-collar workers. *“We have strong rent-seeking networks in the executive branch and government agencies… And these are the main roots of smuggling. The Ministry of Health, Customs, MIMT, and CHCSGC must develop regulatory rules to eliminate golden signatures for issuing import and export licenses”* (p22).

#### Lack of economic stability

Due to the lack of economic stability and the existing worries, we are unable to focus first on production and then on smuggling. From someone perspective: *“I say that the cause of the pharmaceutical crisis in Iran is the president and his first vice-president; the health minister is not to blame; in a country with 50% inflation, can you control the price with decrees? Now, the result is that the producer does not produce…; the price should be adjusted at the appropriate time”. (p1)*

## Monitoring and evaluation

The current evaluation and monitoring system of PSHGs is not working well. The stronger the current control system is, the less useful it becomes. Respondents identified two main issues in this area: Procedures and practices, and Insufficient/workable incentives.

### Procedures and practices

#### Lack of a comprehensive information and statistical system

Interviewees on this topic pointed to the lack of accurate registration and collection of statistics due to Iran’s embargo conditions, the lack of segregation of product groups and related subgroups, delays in registration, and the lack of intersection and sharing of information between organizations. One of the interviewees stated, *“Our country is under embargo and countries that circumvent the embargo are fined based on the approval of the national organization, so accurate registration and seizure are not done”* (p3). According to another, “*Trade information should be officially registered and separated from the base. Separation is also necessary for health goods”* (p8). “…*if we consider their information to be complete and correct, there is still a gap, the registration takes place in the next statistical period” (*p5). *The TTAC system is static, and the Ministry of Health is just a productive system. Now, the systems need to detect errors through the crossing and sharing of information and the monitoring of goods entering and leaving the chain”* (p10).

#### Lack of information transparency

The interviewees highlighted issues such as abuse, monopoly of power, and lack of responsibility in recording information as the main reasons for the lack of transparency. An interviewee remarked: “*What does smuggling mean? It means a lack of transparency”* (p7).*“Transparency and rule-making are the two basic principles of the World Trade Organization. When you are in a glass room and everything is transparent, you can expect that corruption, rent-seeking, and infringements are reduced to a minimum… If data and information can be monitored and different organizations do not want to exploit their systemic rent-seeking, it can make control and monitoring easier*” (p2).

#### Lack of systematic attitude in the monitoring process

We have several environments: economic, political, cultural, and security; if we have a problem in one of these environments, there will be a problem in the surrounding area environment. Managers should look at issues in a macro and systemic manner. one interviewee explained: *“Domestic manufacturing companies do not track whether their manufactured goods are smuggled or not; However, if the economic institution creates an exclusive agency because it is responsible, it helps that this does not happen because the reputation of its brand is endangered and on the other hand, it wants to control the market under its control and make a profit”* (p9).

#### Complexity of monitoring and evaluation

Legal obstacles to monitoring the performance of pharmacies, and the need to implement a selectivity plan due to the high volume of incoming goods were among the issues highlighted by the interviewees. *“The police and the penitentiary are not allowed to enter pharmacies without* FDA *representatives”* (p13*). Another interviewee also noted, “Customs is not able to check 100% of all the goods declared to them; in general, they should do risk management, use their green, yellow, and red routes, and implement the selectivity plan; in some cases, they can check at random, and in between, smuggled goods can be imported” (p2).*

#### Lack of equipment for advanced electronic monitoring

In order to ensure that the speed of trade and the ease of trade is not impaired, the Customs service is obliged to use modern methods in its controls. One participant comments: *“In risk management, we must use new tools; one of these technologies is X-ray; for example, X-ray baggage, X-ray pallets, X-ray trucks, as well as gates that are used for people and a series of controls that must be done inconspicuously… but some customs offices do not have these tools; it is probably due to lack of funds and the provision of cheap and outdated equipment through tenders” (p2).*

#### Difficulty in monitoring advertisement and promotion of products

The discussion on supervision has been challenged by the significant increase in advertising and promotion of products on Internet platforms and the creation of induced demand by doctors. “*Virtualization and the internet are the best and most widespread forms of smuggling; the possibility of leaking warehouses is reduced, selling products is easy, citizen’s rights of are weakened, and if there’s a health problem, the fraudster closes his site or Instagram page and being to work with another one. Unfortunately, most cases are not identified and dealt with until complications occur and consumers start complaining”* (p13).

#### Ignoring the regulatory role of other responsible institutions

Some participants acknowledged that the role of the Consumer and Producer Protection Organization, which oversees the distribution networks, had been weakened. An interviewee stated: “*The Ministry of Health says that I will not allow anyone else to measure my laboratories. I grant you a license, I have supervision in the market, and I manage it myself.”* (p7).

### Surveyors

#### Failure to select and appoint capable and committed checkers

Some interviewees in the audit and inspections pointed to the necessity of improvements in the level of qualification, expertise and skills of inspectors and change in the approaches. *“We have a long history of putting one policeman for every issue and two policemen for every policeman, and we are doing very well in this work compared to the rest of our work! In monitoring and inspection, I believe we are bothering enough; We have to go the roots, we have to stop the origin, we have come to make a balloon that is inflated, thin, and under pressure, we will make 20–30 holes, then we will say, Sir, close the holes with your fingers”. (p11)*

#### Inefficient motivational system

Policies and programs to prevent the smuggling health products should consider creating an incentive system and eliminating the indifference of inspectors and evaluators. One senior executive: *“Deterrence is an obstacle ahead that neither a person can nor wants to do, even if easy ways to evade the law are available” (p. 30).*

## Discussion

Health goods smuggling has turned into a hardly manageable challenge in some countries, especially LMICs. According to World Customs Organization, counterfeit goods account for approximately 7 to 10% of global trade and the revenues from these sales are growing [12]. It seems currently there is no common understanding of counterfeit medicines; none effective mechanism to combat the illegal circulation of counterfeit medicines at national and international level, no productive international cooperation; not a proper exchange of data, and shortage of single database of detected and registered cases of counterfeit medicines, all together which making any cooperation between law enforcement agencies of different countries highly challenging [1]. This study revealed the challenges for prevention of smuggling health goods in Iran, associated mainly with three areas of formulation, implementation and evaluation of anti-smuggling policies.

Such issues as the inefficient restriction policies on health goods import, parallelism in the policy making, the ambiguity and contradictions in the laws and regulations, the low political priority and support for anti-trafficking programs and the lack of anti-monopoly laws were among the most important challenges in the formulation of anti-smuggling policies in Iran. Given the complex, profitable and multi-actor nature of this area, such issues seem unsurprising and developing and operationalizing legal levers is accordingly a complex task, with inherent problems, institutional obstacles and conflicts of interest [20]. Therefore, it requires an integrated system of directions with the joint efforts of international bodies and organizations, various experts. for instance, the priority directions for the introduction of an effective mechanism to combat the distribution of counterfeit medicines is the development of a complex criminalistics methodical investigation [1].

Nevertheless, tightening the restrictions and bans on imported medicines is not an appropriate policy and might only increase imbalances in the domestic economy, monopolies, smuggling and economic corruption [21]. For instance, political pressure to reduce the price of medicines might fuel the market for substandard and counterfeit medicines [22, 23].

Although laws can act as facilitators, the awareness and commitment of lawmakers to introduce appropriate legislative measures that clearly define and recognize counterfeiting of medicines as a different and much more serious crime than counterfeiting of other types of goods should be enhanced [24]. As such, other measures as training for judges, lawyers and the public, publishing the names of offending pharmacies, and emphasizing the role of pharmacists for the public could be practical [25]. In Poland a lack of awareness of the prevalence of counterfeit medicines launched a public awareness campaign [26].

Furthermore, the importance of strengthening the dialogue between the public health and legal/regulatory communities and the development of alternative or complementary solutions, including stronger regulations and non-punitive measures must be highlighted. Smuggling and fraud of health products could result from the improper interaction between societies, economies, and behaviors [20].

As to the anti-smuggling policy implementation, the role of actors, resources, and guaranties were largely influential; low power of law enforcement bodies, trust issues between actors, consumers’ confidence in domestic goods, lengthy process of licensing, the effect of sanctions and golden signature and the poor supply chain performance were the main barriers for preventing smuggling in the country. In fact, the existence of laws is not in itself a guarantee for the prevention of unlawful incidents, as this requires effective implementation of laws, influenced also by the political commitments of governments, resources, and intergovernmental cooperation [27]. Similar studies have besides emphasized that dealing with the entry of counterfeit or substandard healthcare products requires more investment in human resources, infrastructure, stakeholder coordination, and public information [28, 29].

The cooperation between manufacturers and governments is essential to limit the sale, use and the prevalence of substandard and counterfeit medicines [30]. They can be also reduced by a well-enforced ban on the sale of medicines by informal vendors and by increased attention to supplier qualification in the procurement process [31]. As solutions, building confidence in the quality of generic medicines in public health centers, focusing on improving access to medicines and ensuring an adequate supply of good quality, effective, safe, and affordable medicines are mentioned [32]. Pharmacists have an important role to play in strengthening education processes, warning patients about the risks of purchasing medicines from unknown sources (the Internet or unlicensed shops), advising patients and providers on reporting adverse effects and the effectiveness of medicines, and advising health organizations and policymakers on the design and implementation of relevant policies. Purchasers should focus on ensuring the quality of their services, and prescribers and distributors should be more vigilant [33]. Moreover, coordination between law enforcement agencies in dealing with smuggling and governance styles is required [34].

Monitoring and evaluation (M&E) is core to ensure the effective deployment of policies. The challenges herein included those mainly associated with the related procedures and practices and the role of surveyors. The shortage of viable and advanced information systems, incentivizing mechanisms and capable and committed checkers had caused dysfunctioning policies of anti-smuggling policies in the country. Accordingly, the current M&E seemed to be unsuccessful in preventing and combating the smuggling health goods. Similarly, in Pakistan, a country moving rapidly towards an improved regulatory structure, the need to strengthen regulatory systems and analytical laboratories, and build capacity in the overall area of control of substandard and falsified medicines was echoed. Moreover, a comprehensive and long-term vision with an interdisciplinary, open, progressive and evidence-based approach were allegedly required for a successful transition towards a well-regulated system [35]. 

Pharmaceutical tracking and tracing system (PTTS) is used for the security of pharmaceutical supply chains by some countries [36]. Factors influencing the effective implementation of PTTS include stakeholders such as the government and supply chain actors; their awareness, knowledge, and skills; rules and regulations; financial investment; and technical and digital requirements [36]. In Iran, The electronic system for Tracing, Tracking and Authentication Control (TTAC) has been launched since 2013 to manage the currency allocated to medicines, create a suitable platform for gathering information, management of medicine and medical equipment shortages and financial resources, and prevent wastage and misuse of financial support, and control the targeted payment of insurers. Whilst there is a relatively high level of satisfaction with this system within the health sector, the respondents from other organizations complained about the shortcomings of the system and the lack of clear information provided by the MoH and FDA. They argued that the supply chain should be also controlled by other actors and the information gaps between organizations should be addressed. In Turkey, although stakeholders experienced both physical and software problems in the implementation of such programs, the alignment of the incentives of all stakeholders with the power of the government and the freedom of action for adaptation ultimately led to a successful process [37].

## Limitations

This study also suffered from some limitations; including the reluctance of some participants to disclose all information as they were afraid of possible consequences. They were subsequently reassured that their information would be kept fully confidential and their voices were not be recorded in some cases. Further, we gained the participants’ confidence about the importance of the research and its application. Coordination was also very difficult with the key knowledgeable people and decision-makers at high decision-making levels in different organisations which solved by using various communication channels, especially relying on academic correspondence and continuous follow-up.

## Conclusion

Smuggling of health goods is a complicated, sensitive and challenging phenomenon in all countries and accordingly its prevention also needs a multi-faceted, inter-sectoral and comprehensive approach. Policy decisions upon this issue in the country appeared to be as a disjointed puzzles in practice, and the strategic links are either missing at different levels or their importance is neglected. Especially that the political and institutional factors seemingly had a fundamental role in all stages of the formulation, implementation, and evaluation of smuggling prevention policies. Any form of governance to prevent the smuggling of health goods should be strengthened in its three main functions: policy-making (the normative part of governance), facilitation (the provision of appropriate infrastructure) and regulation (government interventions in economic and social activities).

Political discourses with non-political actors; strengthening the existing laws against conflicts of interest by identifying bottlenecks and restricting loopholes that facilitate opportunities for corruption; establishing a viable transparency, M&E and sizeable reward mechanism; and making painful decisions might be considered as key, but initial steps. This article has also sought to benefit from the importance of interdisciplinary analysis, bringing together political scientists, lawyers, economists, statisticians, and health professionals including physicians and pharmacists.

Given the paucity of similar studies and the sensitivity of the area, the identified challenges could lay the groundwork for developing effective and workable solutions for the country and LMICs. In addition, more qualitative studies are required to identify the solutions for and root causes of smuggling health goods mostly in developing countries from different perspectives; especially considering that the range of issues related to the smuggling of health products is very broad, including legal, political, economic, statistical and information, and social and cultural aspects, according to the type of product.

### Supplementary Information

**Supplementary Material 1.**

## Data Availability

No datasets were generated or analysed during the current study.

## Abbreviations

WHO: World Health Organization
PSHGs: Prevent smuggling of medical goods
R&D: Research and development
PTTS: Pharmaceutical tracking and tracing system
MIMT: Ministry of Industry, Mines, and Trade
GSMS: Global Surveillance and Monitoring System
LMICs: Low- and middle-income countries
CHCSGC: Central Headquarters to Combat the Smuggling of Goods and Currency
SF: Substandard and falsified
