# Supplementary material for: The challenges associated with the prevention of smuggling and counterfeiting health goods in Iran
Source: BMC Public Health. 2024 Jun 11;24:1564. doi: 10.1186/s12889-024-18637-0 (PMC11165840; doi:10.1186/s12889-024-18637-0)
Supplement: Supplementary file 1 — Supplementary Material 1. [file 12889_2024_18637_MOESM1_ESM.docx]

**Interview Guide**

| **Job position and organization:** | **work experience:** | **Field of Study:** |
| --- | --- | --- |
| **work experience:** | **Age:** | **Interview date and place:** |
| **Gender** | **education:** | **Interviewee code** |

Please briefly introduce yourself. Describe the activities of your organization and the role it plays in the process of developing and implementing a health-based policy for the prevention of counterfeiting and smuggling.

**Questions:**

1. In general, what is your opinion on the situation of smuggling of health goods in our country? What factors are influencing the counterfeiting and smuggling of health goods?
2. What changes have been made in the approaches related to the policies of preventing the smuggling of health goods in the country?
3. Have these policies and programs achieved their objectives? In what cases have they been a failure?
4. What are the strengths and weaknesses of these policies and programs and the challenges ahead?
5. What influence have individuals, groups, and organizations had on the prevention of health goods smuggling and counterfeiting?
6. What factors, within or outside the health system, contributed to or hindered the emergence of anti-smuggled health goods programs?
7. Do you think the issue of lobbying and informal connections has been considered as much as it should be?
8. In your opinion, what should be the future policy to prevent the smuggling of health goods in Iran and in which direction should it move?

*Express any other point of view that you may have on the challenges, obstacles, and facilitators in relation to the prevention of the smuggling and counterfeiting of health goods in the country.

*Do you have or know of any documents or reports that would be helpful and useful for this research?

*Can you introduce us to other people who are experts and involved in preventing the smuggling of health goods and who should be interviewed?

****Thank you very much for your time and the information you have given us on this matter. ****
